# Supplementary material for: Cardiac MRI in common marmosets revealing age-dependency of cardiac function
Source: Sci Rep. 2020 Jun 23;10:10221. doi: 10.1038/s41598-020-67157-5 (PMC7311402; doi:10.1038/s41598-020-67157-5)
Supplement: Supplementary file 1 — Supplementary Information. [file 41598_2020_67157_MOESM1_ESM.pdf]

Cardiac MRI in common marmosets reveals age-dependency of cardiac function

Amir Moussavi[1,2*], Matthias Mietsch[2,3,4], Charis Drummer[2,5], Rüdiger Behr[2,5], Judith Mylius[1], Susann Boretius[1,2*]

[1]Functional Imaging Laboratory, German Primate Center, Leibniz Institute for Primate Research, Göttingen, Germany

[2]DZHK (German Center for Cardiovascular Research), partner site Göttingen, Germany

[3]Unit of Infection Models, German Primate Center, Leibniz Institute for Primate Research, Göttingen, Germany

[4]Department of Laboratory Animal Science, German Primate Center, Leibniz Institute for Primate Research, Göttingen, Germany.

[5]Platform Degenerative Diseases, German Primate Center, Leibniz Institute for Primate Research, Göttingen, Germany

Supplementary Figure 1: Wall thickening: Both the absolute and fractional wall thickening correlate with the estimated LV-EF (Pearson's r = 0.76 and 0.75 with $p < 0.001$ for both).

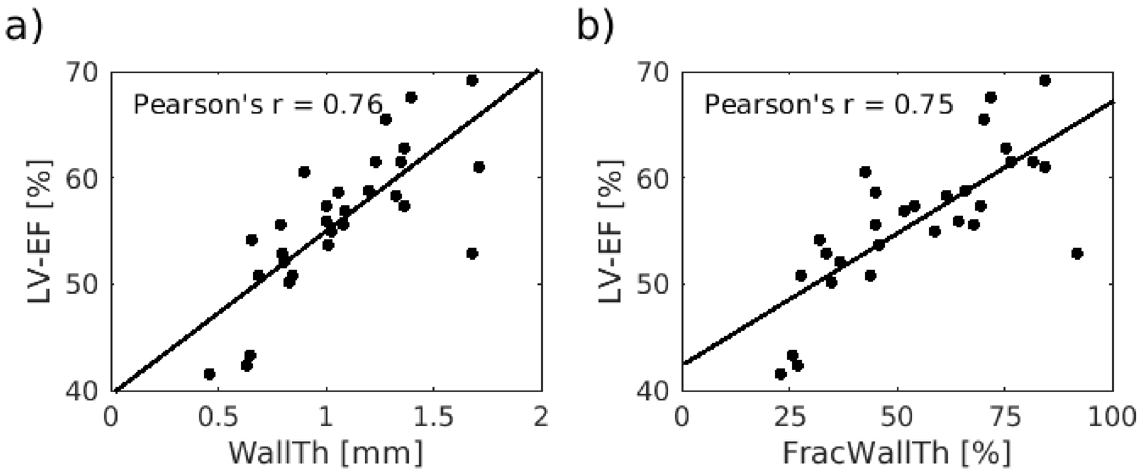

Supplementary Figure 2: Myocardial segmentation. A six sector segmentation model was used to calculate the left ventricular wall thickening of a mid-ventricular slice.

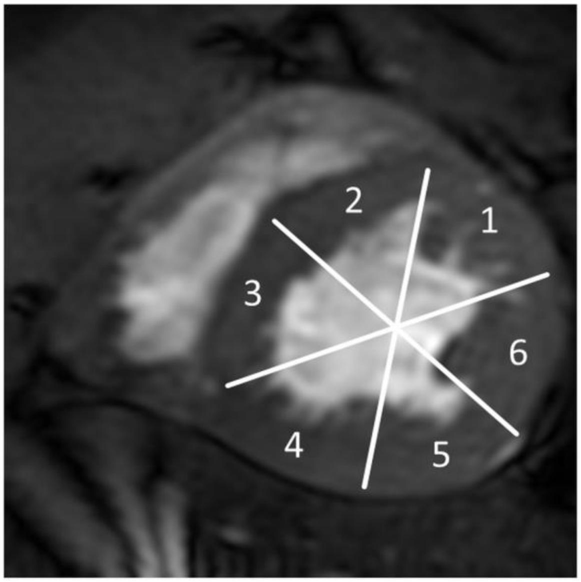

1: Anterior
2: Anteroseptal
3: Inferoseptal
4: Inferior
5: Inferolateral
6: Anterolateral

Supplementary Table: Fractional wall thickening of the left ventricle itemized by segments

FracWallTh	Age groups			Female	Male (all)	All	p-value (one-way ANOVA, age)
	23-58 Months	63-106 Months	110-168 Months				
Anteroseptal	41.0 ± 14.3	40.6 ± 15.6	36.2 ± 21.1	40.9 ± 19.6	37.7 ± 15.0	39.2 ± 17.4	0.82
Inferoseptal	57.4 ± 32.0	56.8 ± 25.5	60.3 ± 17.4	58.5 ± 30.4	57.9 ± 20.3	58.2 ± 25.7	0.96
Inferior	55.1 ± 27.4	52.3 ± 25.4	62.4 ± 18.1	58.3 ± 23.9	55.3 ± 24.5	56.7 ± 24.3	0.67
Inferolateral	73.9 ± 35.6	55.3 ± 30.3	59.9 ± 16.4	62.5 ± 28.0	64.0 ±31.0	63.3 ± 29.6	0.38
Anterolateral	76.0 ± 29.0	55.4 ± 33.1	57.9 ± 26.3	60.0 ± 29.0	66.5 ± 32.2	63.4 ± 30.9	0.29
Anterior	52.8 ± 16.3	46.6 ± 24.3	41.3 ± 18.1	46.5 ± 23.5	47.3 ± 16.7	46.9 ± 20.3	0.47

All data given as arithmetic mean and standard deviation. Significance level $p < 0.05$.
